# Case Report: Penile necrosis associated to paraphimosis with calciphylaxis due to terminal chronic kidney disease

**DOI:** 10.12688/f1000research.18834.1

**Published:** 2019-07-19

**Authors:** J. Antonio Grandez-Urbina, Elizabeth Corrales-Acosta, J. Eduardo Tejeda-Mariaca, Rafael Pichardo-Rodriguez, Herney Garcia-Perdomo

**Affiliations:** 1 Universidad Continental, Calle Junín 355, Miraflores, Lima, 15046, Peru; 2 Urology Deparment, Hospital Alberto Leopoldo Barton Thompson, Avenida Argentina 29, Callao, 07001, Peru; 3 Urology Department, Hospital Nacional Alberto Sabogal Sologuren, Jr. Colina 1081 Bellavista, Callao, 07011, Peru; 4 Biomedical Research Institute, Universidad Ricardo Palma, Avenida Alfredo Benavides 5430, Santiago de Surco, Lima, 15039, Peru; 5 Clinica de Urologia Avanzada Urozen, Avenida Javier Prado Este 3028, San Borja, Lima, 15031, Peru; 6 Medicine School, Universidad del Valle, Calle 4B # 36-00, Cali, Colombia

**Keywords:** Penile necrosis, Calciphylaxis, Nephropathy

## Abstract

**Background**: Penile necrosis is a rare condition that may present in patients with diabetes mellitus or chronic kidney disease (CKD). The recommended treatment is controversial. We report a case of penile necrosis in a diabetic patient caused by episode of paraphimosis associated with uremic arteriopathy treated with partial amputation.

**Clinical Case**: A 53-year-old male with a background of arterial hypertension, diabetes mellitus and CKD in hemodialysis. The patient presented with paraphimosis and glans necrosis. An emergency circumcision was carried out. A doppler ultrasound found fluid collection in the left corpus cavernosum, parietal vascular calcifications and vascular insufficiency in the corpus cavernosum that suggested necrosis. A partial amputation of the penis was carried out. After three years of follow-up, the outcome has remained favorable.

**Conclusions**: Penile necrosis is a rare but serious complication of terminal CKD. In these patients, systemic calciphylaxis is usually observed. The main take-away lesson is that a multidisciplinary approach is necessary.

## Introduction

Calciphylaxis is the process of calcification in small and medium vessels, resulting in necrosis in distal regions of the body such as the lower extremities and the penis, the latter being very infrequent ^1^. This condition may present in patients with diabetes mellitus and/or chronic kidney disease (CKD). It has an incidence of 1–4% in patients with CKD that receive hemodialysis ^2^. Due to the technical advances in hemodialysis and the large number of patients that receive this treatment for long periods of time, the number of cases and risk of calciphylaxis has grown ^3^. Calcific uremic arteriolopathy (CUA), also known as calciphylaxis, is a rare complication of CKD where there is occlusion of microvasculature with mural calcification of the arterioles, causing severe ischemia and necrosis of the tissue ^4^. CUA is a major complication of CKD, which demands timely diagnosis. It carries a high risk of mortality and various complications ^4^.

It is possible to develop ischemic disorders due to arterial calcifications produced by hypercalcemia, as a result of the hyperparathyroidism secondary to CKD ^5^. There are different therapeutic options, but treatment is still controversial. There are no well-established treatment protocols for penile calciphylaxis, and most regimens have been shown to have modest success at best. Treatment options include local wound care, partial or total penectomy, parathyroidectomy, sodium thiosulfate and revascularization ^6, 7^.

On the other hand, paraphimosis occurs when the foreskin of the penis is retracted over the glans and cannot be replaced in its normal position. The tight ring of preputial skin constricts the distal penis causing vascular occlusion and, if not dealt with quickly, can lead to tissue necrosis and partial amputation ^8^. Complications are time related most commonly due to misdiagnosis ^8^. There are few publications related to this uncommon complication.

We report a case of a patient with end stage renal disease (ESRD) that developed calciphylaxis and consequently distal necrosis of the penis and was treated with an amputation, which resulted in a favorable outcome.

## Case report

### Patient information

A 53-year-old, Mestizo patient that works as an accounting assistant was admitted to our hospital in emergency room. The patient had a prior medical history of arterial hypertension, diabetes mellitus type 2 and ESRD, for which the patient had been undergoing hemodialysis for two years prior to the initial consultation. No prior history of any surgical intervention was indicated. The patient presented to the emergency ward with an ulcerative and painful lesion in the glans, which had been present for two weeks.

### Clinical findings

**Figure 1.**
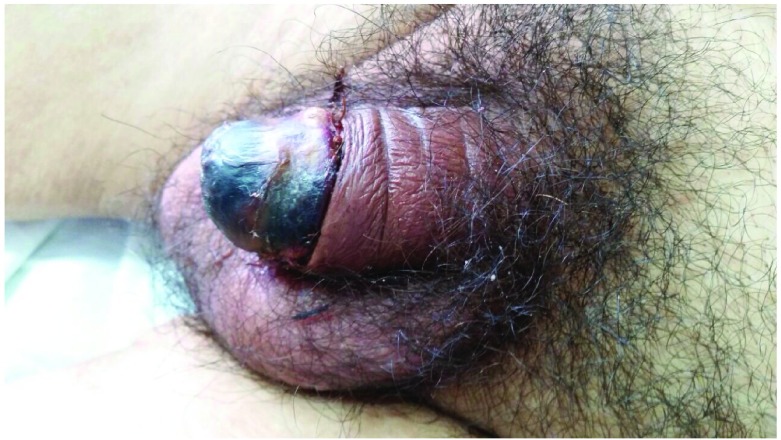
Distal necrosis of penis.

A physical examination revealed foul-smelling distal necrosis of the penis and paraphimosis ( Figure 1) with non-palpable inguinal nodes. No other relevant physical findings were described. The laboratory examination showed elevated creatinine (6.36 mg/dL), urea (114 mg/dL), glucose (119 mg/dL), elevated potassium (5.06 mmol/L), C-reactive protein (8.23 mg/dL) and seric calcium (10.1 mg /dl). On the other hand, hemoglobin (7.9 mg/dL), sodium (134 mmol/L) and albumin (2.9 g/dL) levels were found to be below the normal range. No other significant abnormalities were noted. Doppler ultrasound scans showed fluid collection in the left cavernous body, parietal vascular calcifications and vascular insufficiency in both cavernous bodies, suggestive of penile necrosis. The pathology report confirmed the diagnosis of ischemic penile necrosis due to systemic calciphylaxis.

### Treatment

**Figure 2.**
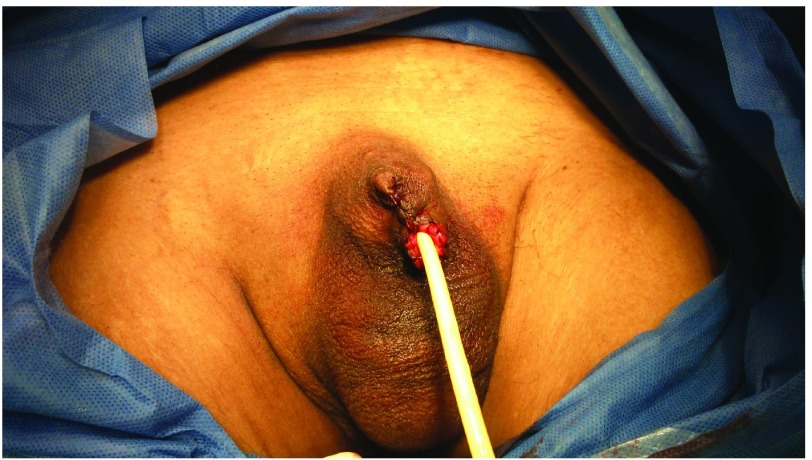
Immediate post-surgical penile partial resection.

The patient was treated with a standard circumcision and resection of the scar in the emergency operating room. Inflammatory tissue was evident but wound dehiscence was not observed in the postoperative period. Subsequently, he received 30 sessions in the hyperbaric oxygen chamber over four weeks. The treatment was carried out at 2.8 absolute atmospheres for a duration of two hours. Broad antibiotic therapy was used in order to reduce the progression of the necrosis (a corrected dose for patients on hemodialysis of 250g Imipenem three times a day) for two weeks. Despite the treatment, the clinical response was not a favorable; we identified by physical examination that the necrotic lesion continued, and the general state of the patient began to worsen. Therefore, in order to avoid a worsening in the infection it was decided that a partial amputation of the penis with preservation of 4cm of penile length would be performed, which had a good outcome, evidencing clinical improvement and a decrease in C-reactive protein ( Figure 2).

### Outcome and follow-up

**Figure 3.**
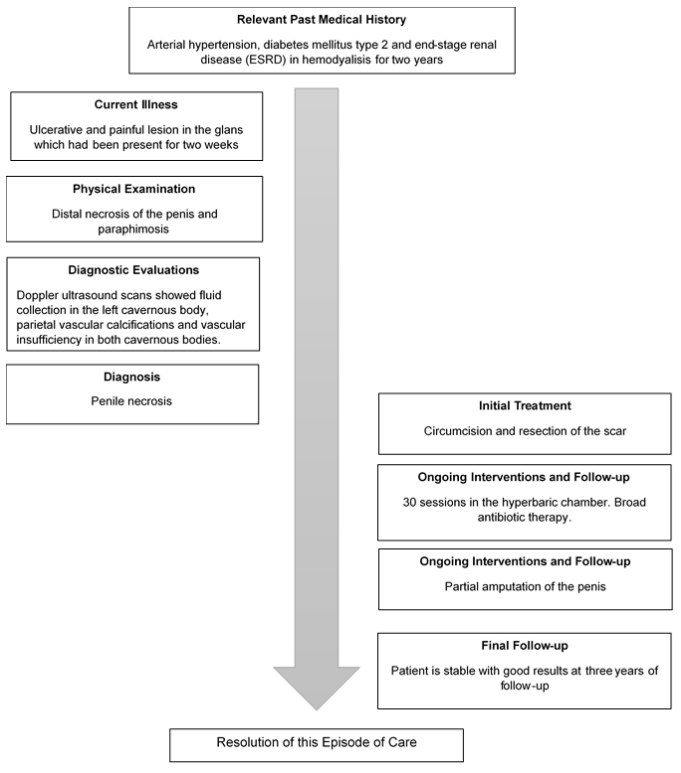
Timeline of the important points in the patient’s medical history, interventions and follow-up.

After three years of follow-up, the patient did not present with urinary symptoms or pain. The patient can void spontaneously. A timeline of the patient’s medical history, interventions and follow-up is shown in Figure 3. 

## Discussion

Penile necrosis in patients with ESRD due to calciphylaxis is infrequent ^9^. In ESRD, the origin of the calciphylaxis is related to secondary hyperparathyroidism, as a result of chronic hyperphosphatemia ^10^. Calciphylaxis results in the total obstruction of the arterial lumen due to arterial wall calcification, producing acute ischemia and necrosis of the affected tissue ^3^. In our case, the arterial irrigation of the penis was affected, producing distal necrosis, an unusual event that is usually avoided due to abundant irrigation and collateral circulation. It represents a poor prognostic sign in ESRD patients and is an indicator of metastatic vascular calcification. Pathogenesis of this life-threatening condition is not clearly understood, and treatment is also controversial ^11^.

The absence of follow-up and treatment with calcitriol for the control of calcium metabolism prior to diagnosis, adding to the absence of treatment for secondary hyperparathyroidism and subsequent calciphylaxis, were the main limitations in this case. However, the post-treatment follow-up and the histological confirmation of the calciphylaxis were important strengths that allowed us to confirm that following the guidelines in previous reports, which recommend starting with the conservative management and following with surgical treatment, were effective and safe with a satisfactory conclusion, despite the high mortality reported for this group of patients.

Penile necrosis produced by calciphylaxis is presented more frequently in patients between 40 and 60 years old. It is associated with ESRD and diabetes mellitus in 100% and 76% of cases respectively, of which our patient had both of risk factors ^3^. Other risk factors obesity, arterial hypertension, use of corticoids, use of inhibitors of vascular calcification and oral anticoagulants ^3^. Our patient had a history of arterial hypertension.

The image studies to perform include doppler ultrasound of the penis, computed tomography (CT) and magnetic resonance imaging (MRI) ^12^. It is suggested that doppler ultrasound is performed as the first line image study to evaluate vascular permeability and blood flow of the penile vessels. CT could be performed secondarily to evaluate with more sensitivity the extension of the vascular calcification into the soft tissues, necrotizing infection of soft tissues and/or ischemia with the presence of air ^12^. MRI scans can identify with more specificity the necrotic borders of the affected tissues ^12^. In our case, we could only perform a doppler ultrasound within the clinical context of the patient. The rest of the image studies could not be performed as it was necessary to rapidly initiate the required treatment.

For the management of penile necrosis there are different therapeutic options, from conservative management to surgical intervention, according to the necessity of the case. Nevertheless, it has a poor prognosis and management is controversial ^9, 12^. In addition to therapy for penile necrosis, it is recommended to initiate treatment for secondary hyperparathyroidism, in order to reduce long term mortality ^6, 13^. In patients undergoing hemodialysis with hyperphosphatemia, it is recommended to use phosphate blockers that do not contain calcium, and in patients with elevated levels of parathormone (PTH), it is recommended to use cinacalcet ^11^. Additionally, studies have reported that necrotic tissue can become a culture medium for multiple microorganisms, so the use of broad-spectrum antibiotics is recommended as a prophylactic measure and empirical therapy ^11^. Empirical antibiotic therapy was used for this patient, as well as surgical treatment, due to the poor response to conservative measures. Treatment for hyperphosphatemia could not be initiated because of the unavailability of the drugs. Karpman *et al.* demonstrated in a case series of 34 patients that survival rate after partial penile amputation and thyroidectomy was superior to partial amputation by itself, with rates of 75% and 28% respectively. The overall mortality was 64%, with a mean time of 2.5 months until death ^6^.

The patient had a favorable recovery after partial penectomy without recurrence of necrosis, despite the failure of the first therapeutic line, even though it has been reported that an increase of vascular flow can improve oxygen transport to the ischemic site ^11, 12^.

## Conclusions

- Penile necrosis constitutes a rare but serious complication associated with ESRD.
- Rapid and timely management of paraphimosis could improve outcomes in patients with multiple co-morbidities.
- For a good clinical outcome, it is necessary to have a high clinical suspicion and to have knowledge of the different elements involved in clinical support.
- A multidisciplinary approach is necessary in the management of this complication.

## Data availability

All data underlying the results are available as part of the article and no additional source data are required.

## Consent

Written informed consent for publication of their clinical details and clinical images were obtained from the patient.
