# Supplementary material for: Case Report: Penile necrosis associated to paraphimosis with calciphylaxis due to terminal chronic kidney disease
Source: F1000Res. 2019 Jul 19;8:1133. [Version 1] doi: 10.12688/f1000research.18834.1 (PMC6685455; doi:10.12688/f1000research.18834.1)
Supplement: Peer review history

# Peer review: Reviewer response for version 1

**DOI:** 10.5256/f1000research.20638.r51408

**Published:** 2019-08-06

**Authors:** Munis M. Ahmed

**Affiliations:** 1 Internal Medicine, Mayo Hospital, King Edward Medical University, Lahore, Pakistan

This is an interesting yet possible explanation for the outcome portrayed. Kindly work on the following fronts to improve the article:

1. Ascertain the word count according to the journal guidelines for case reports.
2. The introduction can be made more concise.
3. Put the lab values in a tabular form.
4. Remove extra headings from the case report section.
5. In the treatment section, put "Broad-spectrum antibiotics" instead of "Broad antibiotics".

I confirm that I have read this submission and believe that I have an appropriate level of expertise to confirm that it is of an acceptable scientific standard.

# Peer review: Reviewer response for version 1

**DOI:** 10.5256/f1000research.20638.r51410

**Published:** 2019-07-30

**Authors:** Félix Grases Freixedas

**Affiliations:** 1 Laboratory of Renal Lithiasis Research, University Institute of Health Sciences Research (IUNICS- IdisBa), University of Balearic Islands, Palma de Mallorca, Spain

The clinical case is well documented and due to its low prevalence it deserves to be indexed.

 The authors indicate that the patient had a favorable recovery after partial penectomy without recurrence of necrosis.

- What has been the follow-up time since the patient had undergone penectomy?
- What measures will be taken to avoid recurrence?
- Why the treatments for  hyperphosphatemia are unavailable?
- Why the use of inhibitors of vascular calcification can be considered as risk factors of calciphylaxis?

I confirm that I have read this submission and believe that I have an appropriate level of expertise to confirm that it is of an acceptable scientific standard.
